# GPR124 regulates hyaloid blood vessel regression and is associated with endothelial-mesenchymal transition

**DOI:** 10.1038/s41598-026-50835-1

**Published:** 2026-04-29

**Authors:** Laura Hannig, Robin Heiden, Süleyman Ergün, Barbara M. Braunger, Mario Vallon

**Affiliations:** 1 Institute of Anatomy and Cell Biology, University of Würzburg, Würzburg, Germany; 2 Present Address: Institute of Neuroanatomy, University Hospital Hamburg-Eppendorf, Hamburg, Germany; 3 Atlas University Research Center (ARC), Istanbul Atlas University, Istanbul, Turkey

**Keywords:** Cell biology, Molecular biology

## Abstract

Transition from the transient hyaloid vasculature to the mature retinal vascular network is a critical step in mammalian eye development. While WNT7B signaling in endothelial cells regulates programmed hyaloid vessel regression, the specific receptors and downstream mechanisms remain poorly defined. Here, we identify GPR124, a ligand-specific co-receptor for WNT7A/B, as a key regulator of this process. Using an endothelial-specific, tamoxifen-inducible *Gpr124* knockout mouse model, we show that loss of GPR124 in hyaloid endothelium markedly impairs postnatal hyaloid vessel clearance. Notably, GPR124 function in the developing eye was highly compartment-specific: although crucial for hyaloid vessel regression, it was dispensable for retinal angiogenesis, despite being expressed in both vascular beds. *Gpr124* deletion led to downregulation of several established WNT target genes in hyaloid endothelial cells, consistent with a role for GPR124 in mediating WNT/β-catenin signaling, as previously shown in brain endothelium. While GPR124 was not required for induction of isolated apoptotic events in hyaloid vessels, it promoted segmental vessel regression, characterized by linear clusters of apoptotic endothelial cells. Single-cell RNA sequencing of hyaloid endothelial cells, combined with immunostaining, revealed transcriptional changes consistent with partial endothelial-mesenchymal transition (EndMT). Our findings identify GPR124 as a key mediator of hyaloid vessel regression and suggest that GPR124-dependent WNT/β-catenin signaling and EndMT may contribute to vascular remodeling during ocular development.

**Supplementary Information:**

The online version contains supplementary material available at 10.1038/s41598-026-50835-1.

## Introduction

**Fig. 1:**
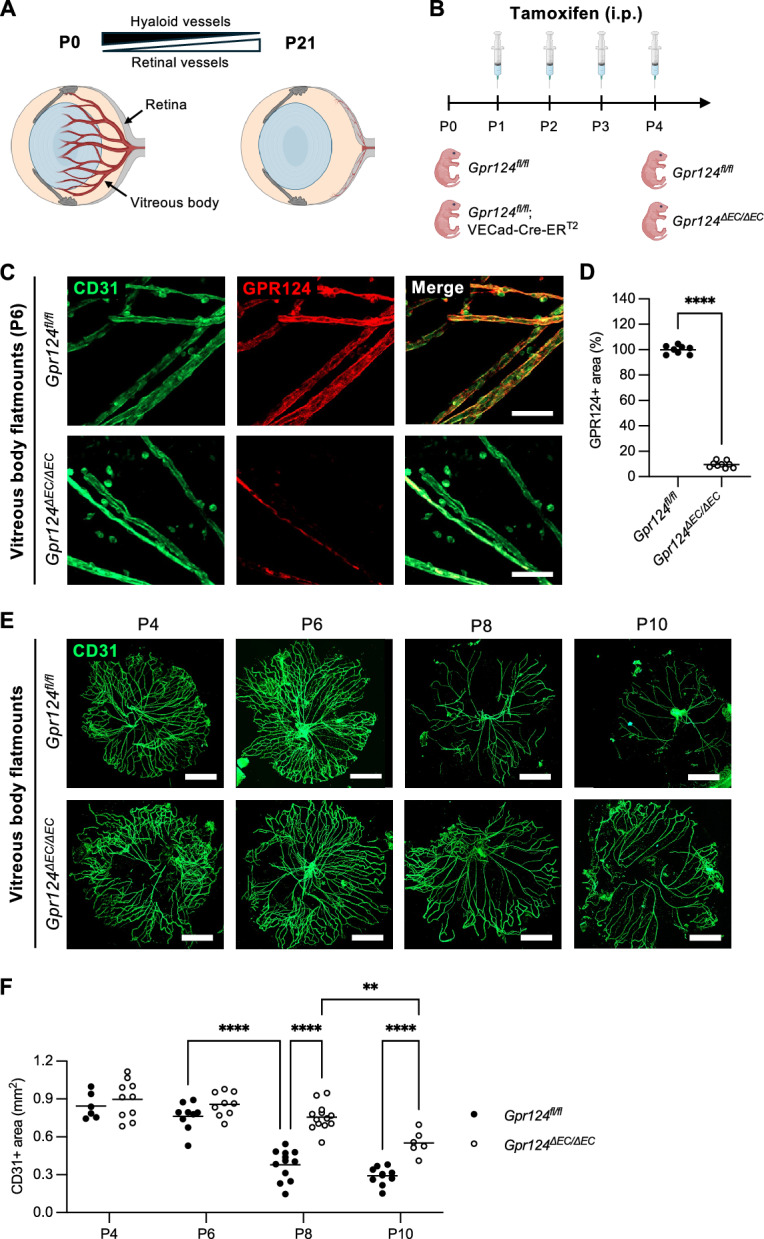
GPR124 regulates hyaloid blood vessel regression. (**A**) Schematic illustrating the timing of hyaloid vessel regression and retinal angiogenesis during mouse eye development (partially created in BioRender. Vallon, M. (2025) https://BioRender.com/oomc7hn). P, postnatal day. (**B**) Schematic of endothelial-specific *Gpr124* knockout induction in *Gpr124fl/fl*; VECad-Cre-ERT2 mice via tamoxifen injections (partially created in BioRender. Vallon, M. (2025) https://BioRender.com/vcpoytw and https://BioRender.com/u5bcw8y). Control littermates (*Gpr124fl/f*) received the same treatment. VECad-Cre-ERT2, vascular endothelial cadherin promoterdriven Cre recombinase fused to a tamoxifen-responsive estrogen receptor. (**C**) CD31 and GPR124 co-immunofluorescence staining of vitreous body flatmounts from P6 *Gpr124*^*fl/fl*^ and *Gpr124*^Δ*EC/*Δ*EC*^ mice. Scale bar: 25 µm. (**D**) Quantification of GPR124 expression in biological replicates of (**C**). The GPR124-positive area per flatmount was normalized to the CD31-positive area and expressed as a percentage of control (*Gpr124fl/fl*). Each dot represents a biological replicate. Horizontal lines represent mean values (n ≥ 8 mice). ****p < 0.0001 (unpaired t-test). (**E**) CD31 immunofluorescence staining of vitreous body flatmounts from *Gpr124*^*fl/fl*^ and *Gpr124*^Δ*EC/*Δ*EC*^ mice at indicated postnatal days. Scale bar: 500 µm. (**F**) Quantification of hyaloid vessel regression (CD31-positive area) in biological replicates of (E). Each dot represents a biological replicate. Horizontal lines represent mean values (n ≥ 6 mice). **p < 0.01, ****p < 0.0001 (two-way ANOVA with Tukey’s post-hoc test). See also Fig. S1.

Hyaloid blood vessels transiently supply the developing vitreous body and lens with oxygen and nutrients^1^. In humans, hyaloid vessel regression starts around the 13th week of gestation, coinciding with the onset of retinal angiogenesis. By birth, this temporary circulatory system usually has fully regressed. In contrast, in mice, hyaloid vessel regression and retinal angiogenesis occur postnatally, with retinal angiogenesis commencing at postnatal day 0 (P0) and hyaloid vessel regression at around P4^2^. Both processes conclude around P21 (Fig. 1A). Dysregulation of hyaloid vessel regression in humans has been linked to persistent hyperplastic primary vitreous (PHPV), a congenital eye disorder that can range from visual impairment to retinal detachment and blindness^3^. However, the mechanisms of pathological persistence and physiological regression of hyaloid vessels remain incompletely understood.

Impaired hyaloid vessel regression is often linked to defective retinal angiogenesis^4–6^, suggesting a crosstalk between these processes. Indeed, it has been reported that retinal angiogenesis induces upregulation of vascular endothelial growth factor receptor 2 (VEGFR2) in retinal neurons, resulting in VEGF sequestration^7^. This leads to a decrease in ocular VEGF levels driving hyaloid vessel regression. Another pathway that has been implicated in hyaloid vessel regression is transforming growth factor β (TGFβ) signaling^8^. Mice with postnatal deletion of TGFβ receptor 2 (*Tgfbr2*) exhibit impaired retinal angiogenesis and persistent hyaloid blood vessels.

WNT/β-catenin signaling in endothelial cells is a key regulator of hyaloid vessel clearance^4,6,9,10^. Hyaloid macrophages, resident macrophages of the vitreous body, postnatally secrete WNT7B activating WNT/β-catenin signaling in hyaloid endothelial cells^9^. It has been suggested that WNT7B signaling triggers initiating apoptotic events in hyaloid endothelial cells resulting in local vessel occlusion, blood flow stasis, and secondary apoptosis and regression of affected vessel segments^9,11^. However, recent evidence challenges this model, suggesting that apoptosis is a consequence, rather than a driver of blood vessel regression^12,13^. Loss of WNT7B signaling, as demonstrated in *Wnt7b* knockout mice, leads to persistent hyaloid vessels^9^. Similarly, deletion of the WNT co-receptors low-density lipoprotein receptor-related protein 5 and/or 6 (*Lrp5/6*) results in impaired hyaloid vessel regression^4,9,10^. Despite these findings, the roles of other WNT receptors, the downstream target genes, and the mechanisms by which WNT/β-catenin signaling induces hyaloid endothelial cell apoptosis remain poorly understood.

Activation of WNT/β-catenin signaling by WNT7A and WNT7B also plays a pivotal role in the developing central nervous system (CNS), where it drives angiogenesis and blood–brain barrier formation^14^. WNT7 signaling in CNS endothelial cells requires classical WNT receptors of the Frizzled and LRP families as well as two additional WNT7-specific co-receptors: the G protein-coupled receptor 124 (GPR124) and the reversion-inducing cysteine-rich protein with Kazal motifs (RECK)^15–17^.

Based on its function in CNS endothelial cells, we hypothesized that GPR124 (also known as TEM5 or ADGRA2) mediates WNT7B signaling in hyaloid endothelial cells to regulate vessel regression. Consistent with this hypothesis, endothelial-specific *Gpr124* knockout mice exhibited defective hyaloid vessel clearance. While scattered apoptotic events in intact hyaloid vessels occurred independently of GPR124, this receptor promoted synchronous apoptosis of endothelial cells across entire vessel segments. Further analyses confirmed that GPR124 mediates WNT/β-catenin signaling in hyaloid endothelial cells and revealed that the GPR124-WNT axis induces pro-apoptotic gene expression as well as partial endothelial-mesenchymal transition (EndMT). EndMT might represent a novel mechanism involved in vascular remodeling of the developing eye.

## Material and methods

### Mice

Mice were housed in a controlled pathogen-free environment under a 12 h/12 h light/dark cycle. *Gpr124*^*fl/*+^ mice were obtained from The Jackson Laboratory (strain B6N.Cg-*Adgra2*^*tm1.1Bstc*/^J, #016881)^18^ and intercrossed to obtain homozygously floxed animals (*Gpr124*^*fl/fl*^). *Gpr124*^*fl/fl*^ mice were then crossed with hemizygous VECad-Cre-ER^T2^ transgenic mice^19^ to obtain *Gpr124*^*fl/fl*^; VECad-Cre-ER^T2^ animals. All mouse experiments were performed in accordance with the German Animal Welfare Act (Tierschutzgesetz), EU Directive 2010/63/EU, as well as the ARRIVE guidelines^20^ and had been approved by the local authorities (Regierung von Unterfranken, animal protocol #RUF-55.2.2–2532-2–1677). Intraperitoneal injections (postnatal day 1–4) and euthanasia by decapitation (postnatal day 4–10) were performed without anesthesia. Intraperitoneal injections in mouse pups are considered minimally invasive procedures causing only transient, mild discomfort and therefore do not require anesthesia according to EU Directive 2010/63/EU and the German Animal Welfare Act. Euthanasia of mouse pups was performed by rapid decapitation without prior anesthesia, an approved method for rodents up to postnatal day 10 that induces immediate loss of consciousness. All procedures were applied consistently across experiments.

### Endothelial-specific Gpr124 knockout induction

For experiments, *Gpr124*^*fl/fl*^ mice were crossed with *Gpr124*^*fl/fl*^; VECad-Cre-ER^T2^ mice to obtain litters with 50% control (*Gpr124*^*fl/fl*^) and 50% conditional knockout (*Gpr124*^*fl/fl*^; VECad-Cre-ER^T2^) pups. Both male and female pups were used for experiments. To induce endothelial-specific *Gpr124* knockout in *Gpr124*^*fl/fl*^; VECad-Cre-ER^T2^ pups, littermates from experimental breeding pairs were injected intraperitoneally with tamoxifen (Biomol #Cay13258) daily from P1 to P4. Increasing doses of tamoxifen (P1: 50 µg, P2/P3: 75 µg, P4: 100 µg) diluted in corn oil (Thermo Fisher Scientific #405,435,000) were injected into the lower right abdomen (50 µl) as previously described^21^. Both control and conditional knockout littermates were injected.

### Vitreous body flatmount preparation and staining

Vitreous body flatmounts were prepared as described by Wang and colleagues^2^. Briefly, pups (P4-P10) were euthanized by rapid decapitation and eyes enucleated and washed in ice-cold PBS. Eyes were fixed in 4% formaldehyde/PBS for 1 h at room temperature and washed again. 100 µl of 5% molten gelatin (SERVA Electrophoresis #22151), warmed to 37 °C, was injected into the vitreous bodies. Injected eyes were placed into a Petri dish and incubated for 1 h on ice. Fibrous, vascular, and nervous tunics as well as the lens were dissected away from the gelatinized vitreous bodies. Vitreous bodies were transferred to glass slides and incubated at 60 °C for 1 h to melt the gelatin and generate flatmounts. Flatmounts were rehydrated in PBS and postfixed with 4% formaldehyde/PBS for 10 min at room temperature. Specimens were washed three times with PBS (5 min each) and incubated with Protease IV (Bio-Techne/ACD #322381) at 40 °C for 20 min. All following steps were performed at room temperature unless specified otherwise. For terminal deoxynucleotidyl transferase dUTP nick-end labeling (TUNEL), the flatmounts were permeabilized with 0.3% Triton X-100 in PBS for 1 h. Subsequently, the tissue was washed three times with PBS (5 min each), followed by labeling apoptotic cells using the Dead-End Fluorometric TUNEL System (Promega #G3250) according to manufacturer’s instructions. Flatmounts were washed three times with PBS (5 min each) and incubated with 5% normal goat serum (Thermo Fisher Scientific #31873) + 0.3% Triton X-100 in PBS (blocking buffer) for 1 h. Specimens were incubated with primary antibodies (Armenian hamster anti-CD31, 1:200, MilliporeSigma MAB1398Z; rabbit anti-GPR124 ectodomain^17^, 1:100; rat anti-F4/80, 1:100, Abcam ab6640; rabbit anti-NG2, 1:100, MilliporeSigma AV5320; rabbit anti-vWF, 1:100, MilliporeSigma AB7356; rabbit anti-ZEB1, 1:100, Cell Signaling Technology #70512; rabbit anti-LEF1, 1:100, Cell Signaling Technology #2230; rabbit anti-VE-Cadherin, 1:500, Abcam ab205336; rabbit anti-Fibronectin, 1:100, Abcam ab199056) in blocking buffer at 4 °C overnight. Flatmounts were washed as before and incubated with secondary antibodies (goat anti-Armenian hamster IgG-AF488, 1:1000, Jackson ImmunoResearch #127-545-160; goat anti-rabbit IgG-Cy3, 1:1000, Dianova/Biozol #112–165-003; goat anti-rat IgG-Cy5, 1:1000, Dianova/Biozol #112-175-143) and DAPI (1 µg/ml, Roche #10236276001) in blocking buffer for 1 h. Specimens were washed as before and cover slips were mounted using Mowiol 4–88 mounting medium (Carl Roth #0713) containing 25 mg/ml DABCO (Carl Roth #0718).

### Retinal flatmount preparation and staining

P5 pups were euthanized by rapid decapitation and eyes enucleated. Eyes were washed in ice-cold PBS and fixed in 4% formaldehyde/PBS at room temperature for 30 min. Whole retinae were dissected and transferred to a 96-well plate (one retina per well) for staining. Retinae were incubated with 5% normal goat serum (Thermo Fisher Scientific #31873) + 0.3% Triton X-100 in PBS (blocking buffer) for 24 h, followed by incubation with an Armenian hamster anti-CD31 antibody (1:1000, MilliporeSigma MAB1398Z) and a rabbit anti-collagen IV antibody (1:500, Bio-Rad #2150-1740) in blocking buffer for 48 h, both at 4 °C. Retinae were washed five times with PBS (20 min each) and incubated with a goat anti-Armenian hamster IgG-AF488 (1:1000, Jackson ImmunoResearch #127–545-160) and a goat anti-rabbit IgG-Cy3 secondary antibody (1:1000, Dianova/Biozol #112-165-003) at 4 °C for 24 h. After washing the tissue as before, retinae were transferred onto slides and radially incised four times at the periphery for flatmounting. Cover slips were mounted as described above.

### Fluorescence image acquisition and quantification

Stained vitreous body and retinal flatmounts were imaged using an Axio Imager.M2 microscope equipped with an ApoTome.2 module (Carl Zeiss Microscopy GmbH). Flatmount overview images were acquired with a 10 × objective using automated tile-scanning and z-stacking. Z-stacks were compressed into single planes by maximum intensity projection and tiles were merged using the ZEN stitching function.

Quantification of CD31-, GPR124-, and collagen IV-positive areas (mm2) as well as F4/80-positive cell counts was performed using the interactive analysis tool of ZEN 3.1 (Carl Zeiss Microscopy GmbH). All other parameters, including fluorescence intensities (LEF1, ZEB1, VE-cadherin), TUNEL puncta counts, radial vascular expansion (collagen IV), and VWF-positive area, were quantified using the Fiji distribution of ImageJ (version 1.54p). In retinal flatmounts, radial vascular expansion was determined manually using the oval selection tool. An oval fully encompassing the vascular plexus (collagen IV staining) was drawn, the major and minor axis lengths recorded, and radial expansion calculated as half of the mean axis length. Retinal vascular density was calculated using the formula: collagen IV-positive area/[(radial expansion)^2^ × π]. In vitreous body flatmounts, regressing vessel segments were manually identified based on the following criteria: vessel segments exhibiting linear clusters of TUNEL puncta and discontinuous CD31 staining, located between two branch points or a branch point and an endpoint.

All other Fiji quantifications were performed using automated, macro-based batch analyses. Cell types were discriminated based on CD31 positivity and nuclear morphology. Cell nuclei were segmented using the IsoData thresholding algorithm on the DAPI channel followed by watershed processing. Nuclei localized within a solid CD31 mask were defined as endothelial, while those outside the mask were categorized as non-endothelial. Non-endothelial nuclei with a circularity index ≤ 0.6 were classified as pericytes. For nuclear markers, background correction was performed using the signal intensity within the CD31 mask excluding the nuclei. For VE-cadherin, background signal was determined from the area outside the CD31 mask. TUNEL puncta were segmented using the Yen thresholding algorithm followed by watershed processing. All automated masks (vessel area, nuclei, cell types, background, TUNEL puncta) were visually validated and staining artifacts or tissue contaminants were manually excluded. Quantifications were performed on overview images of complete flatmounts, except for VE-cadherin, ZEB1, and VWF, which were quantified in three to five independent fields of view (40 × objective) per flatmount. The mean value of these technical replicates was used to represent a single biological replicate. Eyes from the same animal were considered technical replicates.

### Single-cell RNA sequencing analysis (SORT-seq)

P6 littermates (seven *Gpr124*^*fl/fl*^ and nine *Gpr124*^Δ*EC/*Δ*EC*^ pups) were euthanized, and eyes were enucleated and transferred into ice-cold PBS. Vitreous bodies were dissected, pooled by genotype, and transferred into 500 µl ice-cold HBSS (Thermo Fisher Scientific #2020–117). Single-cell suspensions were generated by supplementing the tissues suspensions with 0.05 mg/ml Subtilisin A (MilliporeSigma #P5380) and incubating at 8 °C for 15 min. After digestion, cells were washed twice with ice-cold PBS/2% FCS. The single-cell suspensions were then co-stained on ice for 1 h with anti-mouse CD31-PE-Cy7 (1:200, Thermo Fisher Scientific #25-0311-82), anti-mouse PDGFRB-AF488 (1:100, Thermo Fisher Scientific #53-1402-82), and 7-AAD (1:20, Thermo Fisher Scientific #00-6993-50) in PBS/2% FCS. Cells were washed as before and sorted into 384-well SORT-seq cell capture plates (Single Cell Discoveries) using the BD FACSMelody Cell Sorter (BD Biosciences). Live (7-AAD low), single cells positive for CD31 or PDGFRB were sorted into the plates (one cell per well, two plates per genotype). Sorted cells were processed and sequenced by Single Cell Discoveries using the SORT-seq PLUS protocol. Reads were mapped to the mouse reference genome (GRCm38) and unique molecular identifier (UMI) counts determined using the STARsolo command-line tool (version 2.7.11b). UMI counts were normalized across plates by scaling to the plate with the highest median UMI count. UMI counts as well as original plate and cell identifiers (barcodes) were merged into a single Seurat object using the Seurat R package (version 5.1.0)^22^. Cells with a total UMI count of 1000−8000 and a mitochondrial gene percentage of < 10% were included in downstream analyses. Cells were assigned to clusters based on expression (UMI count > 0) of *Cldn5* (endothelial cells) and *Pdgfrb* (pericytes). Cells expressing neither marker or both markers were excluded from downstream analyses. Log-normalization (scaling factor 10^4^) and scaling of UMI counts as well as t-distributed stochastic neighbor embedding (t-SNE) dimensional reduction were performed using the corresponding Seurat functions. Cluster markers and differentially expressed genes were identified using the FindMarkers function of Seurat and p-value and absolute log2 fold change cutoffs of < 0.05 and > 1, respectively. Cell type and gene ontology (GO) analyses were performed using the enricher and enrichGO functions of the clusterProfiler R package (version 4.12.6)^23^ in combination with the PanglaoDB database^24^ and the org.Mm.eg.db R package (GO Biological Process, source date: 2024–01-17). Inclusion criteria for GO terms were a minimum overlap of five genes and a p-value < 0.01. For figures, redundant and semantically similar GO terms were consolidated into representative terms using the simplify function of clusterProfiler. Endothelial and mesenchymal markers were retrieved from the PanglaoDB cell type “Endothelial cells” and the GO term "epithelial to mesenchymal transition" (GO:0001837), respectively. Additionally, the endothelial-mesenchymal transition marker *Zeb1* was included^25^. WNT signaling- and TGFβ signaling-related genes were obtained by querying the GO terms "Wnt signaling pathway" (GO:0016055) and "transforming growth factor beta receptor superfamily signaling pathway" (GO:0141091), respectively. Pro-apoptotic and anti-apoptotic genes were retrieved from the GO terms "positive regulation of apoptotic process" (GO:0043065) and "negative regulation of apoptotic process" (GO:0043066), respectively.

### Statistical analyses

Statistical analyses were performed using GraphPad Prism (version 10.3.1), the Seurat R package (version 5.1.0), or the clusterProfiler R package (version 4.12.6). Unpaired t-tests (two groups) and 2-way ANOVA (multiple comparisons) were used to compare immunofluorescence signals (GraphPad Prism). Cluster markers and differentially expressed genes were identified using logistic regression followed by a likelihood ratio test (FindMarkers function of Seurat). Hypergeometric tests were performed to identify cell types and GO terms using the enricher and enrichGO functions of clusterProfiler. A p-value < 0.05 was considered statistically significant.

## Results

### GPR124 regulates hyaloid blood vessel regression

To circumvent embryonic lethality associated with germline knockout of *Gpr124*^26^, we used mice with tamoxifen-inducible, endothelial-specific *Gpr124* deletion (*Gpr124*^*fl/fl*^; VECad-Cre-ER^T2^). Postnatal tamoxifen administration to pups resulted in endothelial-specific *Gpr124* knockout (*Gpr124*^*∆EC/∆EC*^, Fig. 1B−D). Cre-negative littermates treated with tamoxifen served as controls (*Gpr124*^*fl/fl*^). GPR124 expression and knockout efficiency in hyaloid endothelial cells were determined by CD31 and GPR124 co-immunostaining of vitreous body flatmounts from P6 *Gpr124*^*fl/fl*^ and *Gpr124*^*∆EC/∆EC*^ mice (Fig. 1C), followed by quantification (Fig. 1D). Co-localization with CD31 confirmed GPR124 expression in hyaloid endothelial cells, while a ~ 90% decrease in GPR124 immunoreactivity demonstrated high knockout efficiency. Residual GPR124 signal was restricted to occasional vessel segments, suggesting incomplete recombination in a small subset of endothelial cells. However, it cannot be fully excluded that part of the remaining signal reflects non-specific staining.

CD31 staining and quantification in vitreous body flatmounts from *Gpr124*^*fl/fl*^ and *Gpr124*^*∆EC/∆EC*^ mice (P4-P10) revealed impaired hyaloid vessel regression in knockout mice, with significant differences in the CD31-positive area observed from P8 onward (Fig. 1E,F). A significant reduction in the hyaloid vascular area between P8 and P10 in *Gpr124*^*∆EC/∆EC*^ mice indicated that vessel regression is delayed but not completely inhibited.

To rule out confounding effects of the VECad-Cre-ER^T2^ transgene, we compared the hyaloid vascular area between P8 wildtype and VECad-Cre-ER^T2^ pups treated with tamoxifen (Fig. S1A). Mere expression and tamoxifen-induced nuclear translocation of Cre-ER^T2^ in hyaloid endothelial cells did not affect hyaloid vessel clearance. Furthermore, since hyaloid macrophages regulate postnatal vessel regression^27^, we quantified F4/80-positive macrophages (Fig. S1B,C). While macrophage numbers in the vitreous body declined postnatally (P4-P10), there was no significant difference between genotypes, indicating that endothelial GPR124 does not modulate hyaloid macrophage proliferation or survival.

### GPR124 does not modulate retinal angiogenesis

**Fig. 2:**
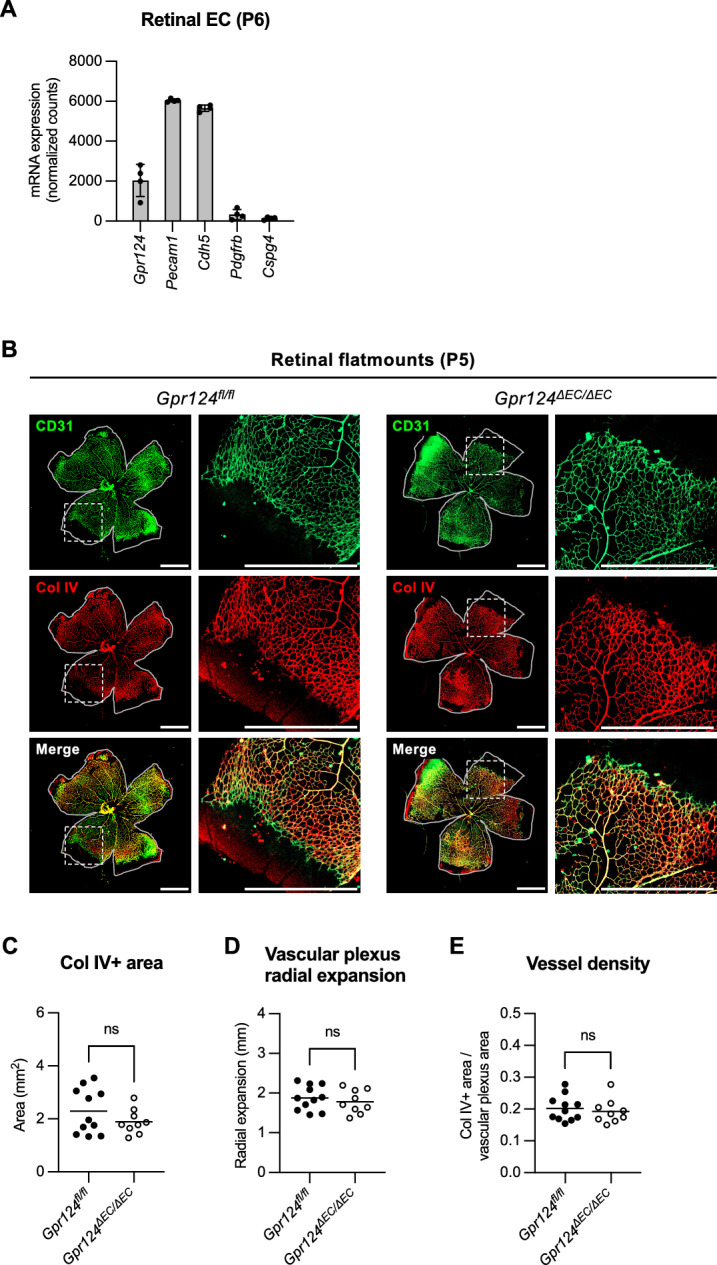
GPR124 does not regulate superficial retinal vascular plexus formation. (**A**) Analysis of published RNA-seq data (GEO: GSE199858) from FACS-isolated retinal endothelial cells (EC) from P6 mice. Bars show mean values ± SD of normalized counts (n = 4 mice). GEO, Gene Expression Omnibus. (**B**) CD31 and collagen IV co-immunofluorescence staining of retinal flatmounts from P5 *Gpr124*^*fl/fl*^ and *Gpr124*Δ*EC/*Δ*EC* mice. Solid gray lines depict retinal leaf borders. Boxed regions are shown at higher magnification on the right. Scale bars: 400 µm. (**C** − **E**) Quantification of retinal angiogenesis in biological replicates of (**B**). Collagen IV-positive vascular area (**C**), radial expansion of the vascular plexus (**D**), and vessel density (**E**) were quantified. Each dot represents a biological replicate. Horizontal lines represent mean values (n ≥ 9 mice). *ns* not significant (unpaired t-test).

Defects in retinal angiogenesis often lead to secondary persistence of hyaloid vessels^4–6^. Since GPR124 is a known regulator of developmental CNS angiogenesis^26^, we investigated its role in retinal vascularization. Analysis of previously published RNA-seq data (GSE199858) revealed that retinal endothelial cells express *Gpr124* at high levels (Fig. 2A). However, CD31 and collagen IV co-immunostaining of P5 retinal flatmounts (Fig. 2B), followed by quantification of the collagen IV-positive vascular area (Fig. 2C), radial expansion of the vascular plexus (Fig. 2D), and vessel density (Fig. 2E), revealed no significant differences between *Gpr124*^*fl/fl*^ and *Gpr124*^*∆EC/∆EC*^ mice. These findings are consistent with a previous report showing that endothelial-specific *Gpr124* deletion does not overtly affect retinal vascular development and blood-retinal barrier formation^28^. Together, these data indicate that GPR124 is dispensable for retinal angiogenesis, highlighting differential requirements for GPR124 across CNS vascular beds. Furthermore, this suggests that GPR124 regulates hyaloid vessel regression directly rather than indirectly by modulating retinal angiogenesis.

### GPR124 regulates WNT target gene expression in hyaloid endothelial cells

**Fig. 3:**
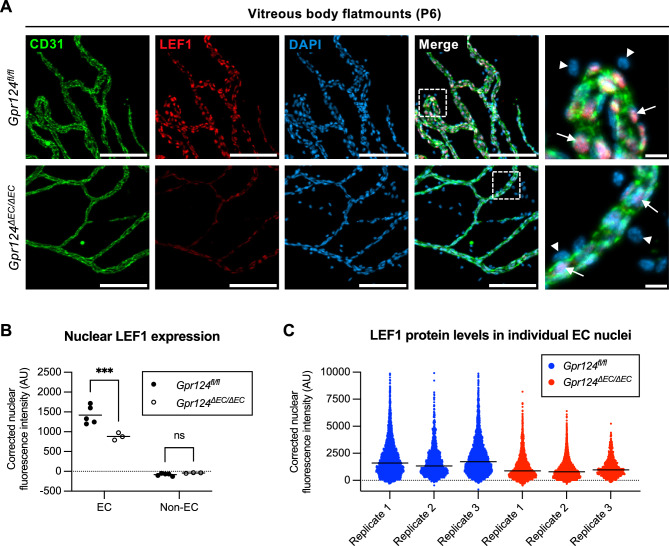
GPR124 regulates WNT target gene expression in hyaloid endothelial cells. (**A**) CD31, LEF1, and DAPI co-staining of vitreous body flatmounts from P6 *Gpr124*^*fl/fl*^ and *Gpr124*^Δ*EC/*Δ*EC*^ mice. Boxed regions are shown at higher magnification on the right. Arrowheads denote non-endothelial cell nuclei. Arrows indicate endothelial cell nuclei. Scale bars: 100 µm (overview); 10 µm (zoom). LEF1, lymphoid enhancer-binding factor 1. (**B**) Quantification of nuclear LEF1 expression in endothelal cells (EC) and non-EC in biological replicates of (**A**). Dots represent median nuclear LEF1 fluorescence intensity per flatmount (background-corrected integrated intensity). Horizontal lines show mean values (n ≥ 3 mice). ***p < 0.001; *ns* not significant (two-way ANOVA with Šídák’s post-hoc test). (**C**) Scatter dot plot depicting LEF1 expression in individual endothelial cell nuclei in biological replicates of (**A**). Dots represent LEF1 fluorescence intensity per nucleus (background-corrected integrated intensity). Horizontal lines show median values.

GPR124 is a key regulator of WNT/β-catenin signaling and WNT target gene expression in CNS endothelial cells^16^. To assess if GPR124 exerts similar control in hyaloid endothelial cells, we co-stained vitreous body flatmounts from P6 *Gpr124*^*fl/fl*^ and *Gpr124*^*∆EC/∆EC*^ mice for CD31 and the WNT target gene LEF1 (Fig. 3A). Nuclear expression of LEF1 in endothelial cells was downregulated by ~ 50% in *Gpr124*^*∆EC/∆EC*^ mice, whereas LEF1 was undetectable in non-endothelial cells of either genotype (Fig. 3B). Single-nucleus analysis of LEF1 expression revealed that *Gpr124* knockout affects WNT target gene expression in virtually all hyaloid endothelial cells (Fig. 3C).

### GPR124 promotes segmental regression of hyaloid vessels

**Fig. 4:**
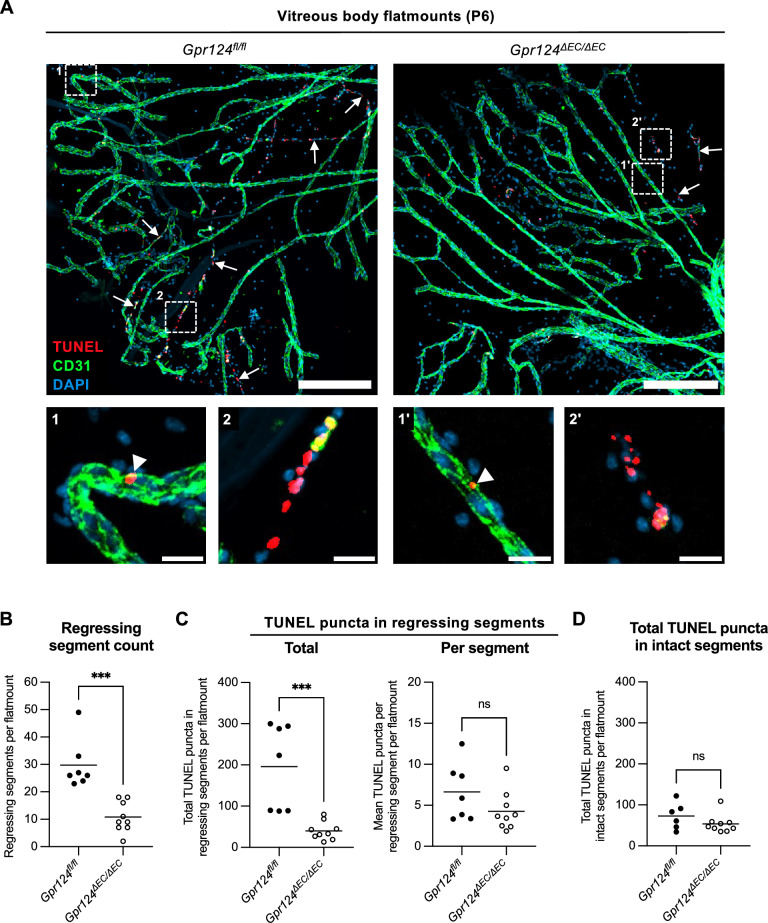
GPR124 regulates segmental regression of hyaloid vessels. (**A**) TUNEL, CD31, and DAPI co-staining of vitreous body flatmounts from P6 *Gpr124*^*fl/fl*^ and *Gpr124*Δ*EC/*Δ*EC* mice. Top: arrows indicate regressing vessel segments, defined by linear clusters of TUNEL puncta and discontinuous CD31 signal. Boxed regions are shown at higher magnification below. Bottom: (1) and (1') show intact vessel segments containing isolated TUNEL-positive cells (arrowheads). (2) and (2') show regressing vessel segments at higher magnification. Scale bars: 200 µm (overview); 20 µm (zoom). TUNEL, terminal deoxynucleotidyl transferase dUTP nick-end labeling. (**B** − **D**) Quantification of regressing vessel segments and TUNEL puncta in biological replicates of (**A**). Each dot represents a biological replicate. Horizontal lines represent mean values (n ≥ 7 mice). ***p < 0.001; *ns* not significant (unpaired t-test). (**B**) Number of regressing vessel segments per flatmount. A regressing segment was defined by linear clusters of TUNEL puncta and discontinuous CD31 signal, located between two branch points or a branch point and an endpoint. (**C**) TUNEL puncta in regressing vessel segments. Left: total puncta in regressing segments per flatmount; right: mean number of puncta per segment. (**D**) Total TUNEL puncta in intact vessel segments per flatmount.

Previous studies suggested that WNT/β-catenin signaling in hyaloid endothelial cells triggers isolated apoptotic events^9,11,27^. These events lead to localized vessel occlusion and blood flow stasis, which in turn induces synchronous secondary apoptosis of endothelial cells within the affected vessel segment. To test if GPR124 mediates vessel regression by a similar mechanism, we performed TUNEL and CD31 co-staining of P6 vitreous body flatmounts (Fig. 4A). Regressing vessel segments were identified by linear clusters of TUNEL-positive cells, collapsed lumina, and discontinuous CD31 signal. Quantification revealed a reduction in the number of regressing segments upon *Gpr124* deletion (Fig. 4B). This decrease correlated with a reduced total number of TUNEL puncta in regressing segments per flatmount (Fig. 4C, left panel). However, the mean number of TUNEL puncta per regressing segment remained unaffected (Fig. 4C, right panel). We also observed isolated apoptotic events (TUNEL puncta) in morphologically intact vessel (Fig. 4A, bottom panels), which have been proposed to initiate segmental regression^9,11,27^. However, the frequency of these events did not differ between genotypes (Fig. 4D). Together, these findings suggest that GPR124 promotes segmental regression of hyaloid vessels by mechanisms other than triggering isolated apoptotic events.

### GPR124 is associated with partial endothelial-mesenchymal transition

**Fig. 5:**
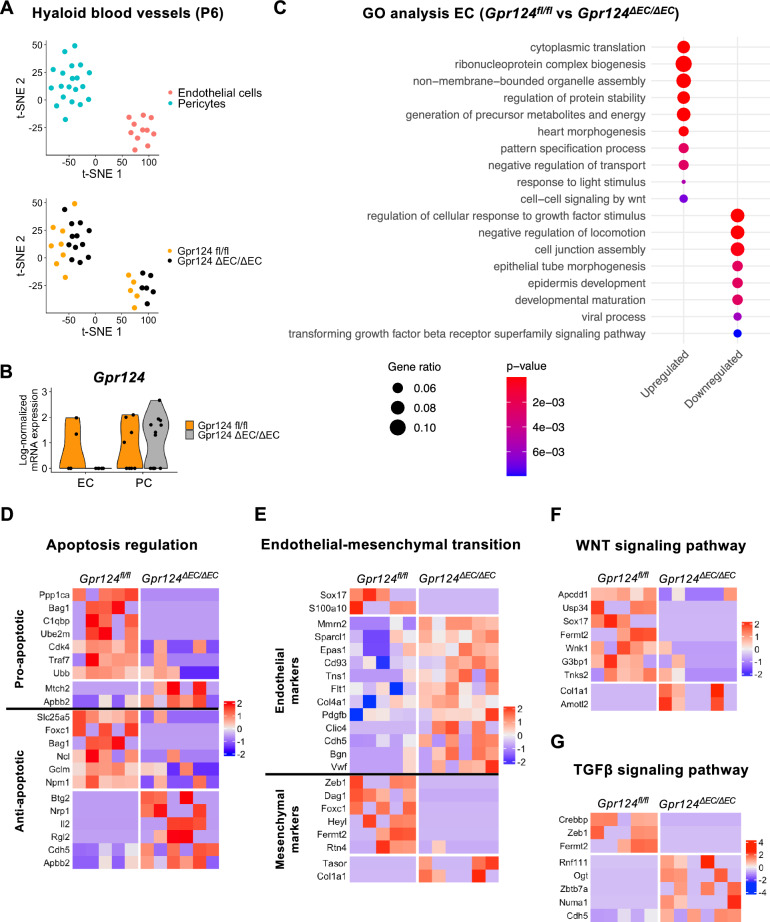
Single-cell RNA sequencing analysis of P6 hyaloid blood vessels. (**A**) t-SNE plots showing clustering of cell types (top) and distribution of genotypes (bottom) for the sequenced cells. t-SNE, t-distributed stochastic neighbor embedding. (**B**) Violin plot depicting log-normalized mRNA expression (UMI counts) of *Gpr124* in endothelial cells (EC) and pericytes (PC) split by genotype. Dots represent individual cells. UMI, unique molecular identifier. (**C**) GO Biological Process analysis of differentially expressed genes in endothelial cells (*Gpr124*^*fl/fl*^ vs *Gpr124*Δ*EC/*Δ*EC*). Differential expression criteria: absolute log2 fold change > 1 and p < 0.05 (logistic regression with likelihood ratio test). Upregulated and downregulated genes were analyzed separately (columns). Redundant and semantically similar terms were consolidated. Complete results are provided in Table S2. Only terms with a minimum overlap of five genes and a p < 0.01 (hypergeometric test) are shown. Gene ratio = overlapping genes: input genes. GO, gene ontology. (**D** − **G**) Heatmaps depicting differentially expressed genes (scaled UMI counts) in endothelial cells (*Gpr124*^*fl/fl*^ vs *Gpr124*^Δ*EC/*Δ*EC*^) related to apoptosis regulation (**D**), endothelial-mesenchymal transition (**E**), WNT signaling (**F**), and TGFβ signaling (**G**). Differential expression criteria: absolute log2 fold change > 1 and p < 0.05 (logistic regression with likelihood ratio test). Red = upregulated, blue = downregulated or not detected. See also Fig. S2 and Tables S1−S3.

To elucidate the molecular mechanisms underlying GPR124-mediated hyaloid vessel regression, we performed single-cell RNA sequencing of P6 hyaloid vessels (Fig. 5 and S2). After quality control filtering, 31 cells were retained for downstream analyses (Fig. S2B). t-SNE clustering identified two cell populations: endothelial cells (5 *Gpr124*^*fl/fl*^, 6 *Gpr124*^*∆EC/∆EC*^) and pericytes (10 *Gpr124*^*fl/fl*^, 10 *Gpr124*^*∆EC/∆EC*^) (Fig. 5A and S2C−E, Table S1). While *Gpr124*^*fl/fl*^ and *Gpr124*^*∆EC/∆EC*^ cells did not form distinct clusters, they separated within each cell population. Analysis of *Gpr124* mRNA levels across cell types and genotypes confirmed the knockout and revealed expression in both hyaloid endothelial cells and pericytes (Fig. 5B). Comparative analysis of *Gpr124*^*fl/fl*^ vs *Gpr124*^*∆EC/∆EC*^ hyaloid endothelial cells identified 249 differentially expressed genes (DEG) (Fig. S2F, Table S2). Gene ontology (GO) analysis of genes upregulated in *Gpr124*^*fl/fl*^ cells indicated enrichment of terms associated with translation, ribosome biogenesis, energy metabolism, development, and WNT signaling (Fig. 5C, Table S2). Conversely, downregulated genes were enriched for GO terms related to growth factor response, inhibition of cell migration, cell junction assembly, development, and TGFβ signaling.

While not identified as a major biological process regulated by GPR124, two of the 187 enriched GO terms were related to apoptosis (Table S2). Upon screening the DEG for overlaps with the GO terms "positive regulation of apoptotic process" (GO:0043065) and "negative regulation of apoptotic process" (GO:0043066), 20 genes were identified (Fig. 5D). Interestingly, most pro-apoptotic genes were upregulated in *Gpr124*^*fl/fl*^ cells, whereas negative regulators of apoptosis showed a balanced up- and downregulation.

Notably, 22 DEG were known endothelial or mesenchymal markers, exhibiting an expression pattern consistent with GPR124-dependent endothelial-mesenchymal transition (EndMT)^25^. In *Gpr124*^*fl/fl*^ endothelial cells, multiple endothelial markers were downregulated, and mesenchymal markers were upregulated (Fig. 5E). While our GO analysis did not specifically identify EndMT, the related GO terms "epithelial to mesenchymal transition" (GO:0001837) and "mesenchymal cell differentiation" (GO:0048762) were significantly enriched in the upregulated gene set (Table S2). Consistent with the LEF1 immunostaining, the WNT target genes *Apcdd1* and *Sox17* showed upregulation in *Gpr124*^*fl/fl*^ cells, along with several other WNT signaling regulators (Fig. 5F). As indicated by the GO analysis, several genes associated with TGFβ signaling were also differentially expressed between *Gpr124*^*fl/fl*^ and *Gpr124*^*∆EC/∆EC*^ endothelial cells (Fig. 5G).

**Fig. 6:**
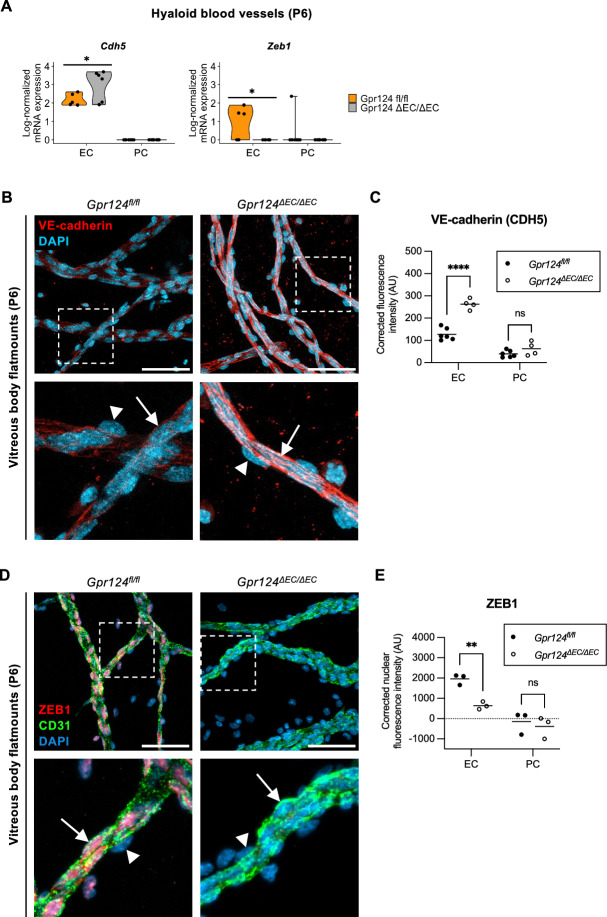
GPR124 is associated with partial endothelial-mesenchymal transition in hyaloid endothelial cells. (**A**) Violin plots depicting log-normalized mRNA expression (UMI counts) of *Cdh5* and *Zeb1* in hyaloid endothelial cells (EC) and pericytes (PC) from P6 mice (*Gpr124*^*fl/fl*^ vs *Gpr124*^Δ*EC/*Δ*EC*^). Dots represent individual cells. *p < 0.05 (logistic regression with likelihood ratio test). Exact p-values are provided in Table S2. UMI, unique molecular identifier; *Cdh5*, cadherin 5; *Zeb1*, zinc finger Ebox-binding homeobox 1. (**B**) VE-cadherin (CDH5) and DAPI co-staining of vitreous body flatmounts from P6 *Gpr124fl/fl* and *Gpr124*Δ*EC/*Δ*EC* mice. Boxed regions are shown at higher magnification below. Arrows denote endothelial cells; arrowheads indicate pericytes. Scale bar: 50 µm. (**C**) Quantification of VE-cadherin (CDH5) expression in biological replicates of (**B**). Dots represent VE-cadherin fluorescence intensity per flatmount (background-corrected mean intensity). Horizontal lines show mean values (n ≥ 4 mice). ****p < 0.0001; ns, not significant (two-way ANOVA with Šídák’s post-hoc test). (**D**) ZEB1, CD31, and DAPI co-staining of vitreous body flatmounts from P6 *Gpr124fl/fl* and *Gpr124*^Δ*EC/*Δ*EC*^ mice. Boxed regions are shown at higher magnification below. Arrows denote endothelial cells; arrowheads indicate pericytes. Scale bar: 50 µm. (**E**) Quantification of ZEB1 expression in biological replicates of (**D**). Dots represent median nuclear ZEB1 fluorescence intensity per flatmount (background-corrected integrated intensity). Horizontal lines show mean values (n = 3 mice). **p < 0.01; ns, not significant (two-way ANOVA with Šídák’s post-hoc test). See also Fig. S4 and Table S2.

To validate differential expression of EndMT markers at the protein level, we performed immunostaining of vitreous body flatmounts from P6 *Gpr124*^*fl/fl*^ and *Gpr124*^*∆EC/∆EC*^ mice (Fig. 6 and S3). Downregulation of the endothelial markers *Cdh5* (VE-cadherin) and *Vwf* at the mRNA level (Fig. 6A and S3A) correlated with downregulation at the protein level (Fig. 6B,C and S3B-C). Conversely, upregulation of the mesenchymal marker *Zeb1*^25^ at the mRNA level (Fig. 6A) was confirmed at the protein level (Fig. 6D,E).

Although not directly affected by the *Gpr124* knockout, hyaloid pericytes displayed significant transcriptional changes in *Gpr124*^*fl/fl*^ vs *Gpr124*^*∆EC/∆EC*^ mice, with 327 upregulated and 213 downregulated genes (Fig. S2G, Table S3). GO analysis revealed association of the differentially expressed genes with protein degradation, development, apoptosis, and autophagy (Fig. S3H, Table S3), suggesting a secondary stress response in hyaloid pericytes.

### Hyaloid endothelial cell apoptosis precedes pericyte loss

Pericytes are essential regulators of blood vessel integrity^29^ and could potentially orchestrate hyaloid vessel regression. GPR124-mediated signaling in endothelial cells might trigger pericyte loss, leading to subsequent vessel destabilization and secondary endothelial cell apoptosis. To investigate the temporal sequence of endothelial and pericyte loss during hyaloid vessel clearance, we performed CD31 and NG2 co-immunostaining of vitreous body flatmounts at different postnatal days (Fig. S4A). Regressing vessel segments exhibited apoptotic endothelial cells, evidenced by fragmented and condensed CD31 signal, while the surrounding NG2-positive pericytes appeared non-apoptotic and interconnected. This indicates that endothelial cell apoptosis precedes pericyte loss during hyaloid vessel regression.

To assess the fate of pericytes in hyaloid vessel clearance, we performed CD31 and fibronectin co-immunostaining of vitreous body flatmounts (Fig. S4B). This staining identified a late stage of regression characterized by acellular “ghost vessels”. These structures consisted merely of residual basement membrane strands devoid of both endothelial cells and pericytes, indicating that pericytes eventually undergo apoptosis or migrate away following endothelial cell death.

## Discussion

In this study, we identify GPR124 as a key regulator of hyaloid blood vessel regression. Our findings are consistent with a model in which GPR124 promotes WNT/β-catenin signaling and partial endothelial-mesenchymal transition (EndMT) in hyaloid endothelial cells.

In the developing CNS, WNT7A and WNT7B secreted by neuroepithelial cells activate β-catenin signaling in endothelial cells to induce angiogenesis and formation of the blood–brain barrier^14^. In contrast, in the developing vitreous body, WNT7B released by macrophages activates β-catenin signaling in endothelial cells to mediate hyaloid vessel regression^9^. These divergent functions underscore the context-dependent nature of WNT signaling, likely reflecting differences in cellular environment, pathway crosstalk, and downstream transcriptional programs. While WNT7A/B signaling has been extensively studied in CNS endothelial cells, the specific receptors and downstream mechanisms of WNT7B signaling in hyaloid endothelial cells have remained poorly defined.

GPR124 is a well-established WNT7A/B co-receptor in CNS endothelial cells^16,17,30^. While not specifically investigated in this study, our data are consistent with a model in which GPR124 mediates WNT7B signaling in hyaloid endothelial cells. This is supported by the similarity between the *Gpr124* knockout phenotype observed here and previously reported *Wnt7b* deficiency^9^. Furthermore, reduced expression of WNT target genes, including *Lef1*, *Apcdd1*, and *Sox17*, in *Gpr124*-deficient hyaloid endothelial cells is consistent with attenuated WNT/β-catenin signaling. However, we cannot entirely exclude that the observed phenotype was caused by dysregulation of other pathways. Rescue experiments using genetic activation of β-catenin (e.g., exon 3 deletion) will be required to definitively establish causality.

Endothelial-specific *Gpr124* deletion impaired hyaloid blood vessel regression but did not completely block it. Residual vessel regression may reflect contributions from GPR124-independent WNT ligands or complementary pathways such as VEGF and TGFβ signaling^7,8^. Although expression of LEF1 was significantly downregulated in *Gpr124* knockout hyaloid endothelial cells, substantial residual expression (~ 50%) suggests persistent WNT/β-catenin activity. Norrin, a soluble WNT agonist expressed in Müller glia and released into the retina and vitreous, is known to activate WNT/β-catenin signaling in retinal endothelial cells^31^ and may contribute to this residual signaling. Furthermore, based on our immunostaining, ~ 10% of *Gpr124* knockout endothelial cells appear to be non-recombined, potentially contributing to the observed residual WNT signaling and vessel regression.

LEF1 expression was undetectable in CD31-negative hyaloid cells, which likely include pericytes and macrophages. This may reflect either low or absent WNT/β-catenin signaling activity in these cells, or LEF1 expression below detection limits. Prior studies proposed that macrophage-derived WNT7B acts as a short-range signal, activating WNT/β-catenin in only a subset of hyaloid endothelial cells^9,11,27^. Our data, however, suggest that disruption of the GPR124-WNT7B axis broadly affects WNT target gene expression across nearly all hyaloid endothelial cells. This apparent discrepancy may reflect differences in sensitivity and readout of pathway activity (LEF1 immunostaining vs β-catenin reporters).

Interestingly, while GPR124 was not required for induction of isolated apoptotic events in hyaloid vessels, it promoted segmental vessel regression, characterized by linear clusters of apoptotic endothelial cells. Segmental vessel regression is typically associated with disrupted blood flow and local hemostasis, which can trigger endothelial apoptosis^13,32^. Single-cell RNA sequencing suggested GPR124-dependent upregulation of pro-apoptotic genes in hyaloid endothelial cells, although it remains unclear whether this represents a primary driver or secondary consequence of vessel clearance. In fact, apoptosis has been proposed to be a downstream event rather than an initiator of vessel regression^12,13^.

A key limitation of our single-cell RNA sequencing (SORT-seq) data is the low number of cells retained after quality control. While stringent filtering ensured analysis of intact cells, it reduced statistical power and necessitates cautious interpretation. Consequently, transcriptomic findings should be considered indicative rather than definitive and require validation by independent approaches. We therefore complemented these analyses with immunostaining of selected targets. Nevertheless, false positive or negative differential expression cannot be fully excluded. The high fraction of cells failing quality control likely reflects technical challenges associated with cell dissociation and/or sorting. While relaxed filtering thresholds increased cell numbers, they introduced substantial noise and impaired clustering and differential expression analysis. We therefore prioritized stringent quality control to ensure robustness of the retained dataset.

Our transcriptomic analyses, together with immunostaining, are consistent with features associated with partial EndMT, a process by which endothelial cells lose their characteristic markers and functions, while acquiring mesenchymal traits, such as enhanced motility and extracellular matrix production^25^. EndMT contributes to diverse physiological and pathological processes (e.g., cardiac valvulogenesis, atherosclerosis, fibrosis), with TGFβ signaling as a core driver, often acting in concert with WNT, Notch, or Endothelin signaling^25^. Our data indicate that most hyaloid endothelial cells undergo incomplete EndMT rather than full transdifferentiation, retaining endothelial markers such as CD31 until at least P10. Whether a small subset of the cells fully transitions, remains to be determined. Transcriptomic changes including upregulation of ribosomal protein genes and downregulation of migration inhibitors further support an EndMT signature^25,33,34^. Upregulation of *Zeb1* and downregulation of *Cdh5* (VE-cadherin), both TGFβ target genes and key inducers of EndMT^25^, suggest potential cooperation between WNT and TGFβ signaling, aligning with prior evidence of their synergy^35–37^.

GPR124-dependent partial EndMT might contribute to hyaloid vessel regression. By downregulating key endothelial junction proteins and promoting a mesenchymal-like state, it may compromise the structural and anticoagulant integrity of the hyaloid endothelium. This transition might induce local blood flow stasis required to trigger the observed segmental regression of hyaloid vessels. This hypothesis is further supported by studies reporting that suppression of endothelial identity (by ETS transcription factor inhibition) accelerates hyaloid vessel clearance^38^.

Endothelial GPR124 also induced a transcriptional response in hyaloid pericytes, including genes associated with protein degradation, apoptosis, and autophagy. Overall, these transcriptional changes indicate a stress response potentially driven by endothelial cell apoptosis, blood flow stasis, ischemia, or downregulation of paracrine survival signals (e.g. *Pdgfb*). Immunostaining revealed sequential loss of cell types during hyaloid vessel clearance with endothelial cell apoptosis preceding pericyte loss.

This study advances our understanding of the molecular mechanisms underlying hyaloid vessel regression and extends vascular regulation by GPR124 beyond the CNS. The association of EndMT with physiologic blood vessel regression may be conserved across other vascular beds and could serve as a therapeutic target for treating disorders such as persistent hyperplastic primary vitreous (PHPV).

## Supplementary Information

Supplementary Information 1.

Supplementary Information 2.

Supplementary Information 3.

Supplementary Information 4.

## Data Availability

Raw and processed single-cell RNA sequencing data have been deposited in Gene Expression Omnibus (GEO) under accession number [GSE284257] (https:/www.ncbi.nlm.nih.gov/geo/query/acc.cgi?acc=GSE284257).

## References

[1] Lutty, G. A. & McLeod, D. S. Development of the hyaloid, choroidal and retinal vasculatures in the fetal human eye. *Prog. Retin. Eye Res.* **62**, 58–76 (2018). 29081352 10.1016/j.preteyeres.2017.10.001 PMC5776052

[2] Wang, Z., Liu, C. H., Huang, S. & Chen, J. Assessment and characterization of hyaloid vessels in mice. *J. Vis. Exp.* **147**, 10–3791 (2019). 10.3791/59222 PMC7028315 31157789

[3] Silbert, M. & Gurwood, A. S. Persistent hyperplastic primary vitreous. *Clin. Eye Vis. Care* **12**, 131–137 (2000). 11137427 10.1016/s0953-4431(00)00054-0

[4] Chen, J. et al. Retinal expression of Wnt-pathway mediated genes in low-density lipoprotein receptor-related protein 5 (Lrp5) knockout mice. *PLoS ONE* **7**, e30203 (2012). 22272305 10.1371/journal.pone.0030203 PMC3260226

[5] Gale, N. W. et al. Angiopoietin-2 is required for postnatal angiogenesis and lymphatic patterning, and only the latter role is rescued by Angiopoietin-1. *Dev. Cell* **3**, 411–423 (2002). 12361603 10.1016/s1534-5807(02)00217-4

[6] Ohlmann, A. V., Adamek, E., Ohlmann, A. & Lutjen-Drecoll, E. Norrie gene product is necessary for regression of hyaloid vessels. *Invest. Ophthalmol. Vis. Sci.* **45**, 2384–2390 (2004). 15223821 10.1167/iovs.03-1214

[7] Yoshikawa, Y. et al. Developmental regression of hyaloid vasculature is triggered by neurons. *J. Exp. Med.* **213**, 1175–1183 (2016). 27325890 10.1084/jem.20151966 PMC4925022

[8] Braunger, B. M. et al. Deletion of ocular transforming growth factor beta signaling mimics essential characteristics of diabetic retinopathy. *Am. J. Pathol.* **185**, 1749–1768 (2015). 25857227 10.1016/j.ajpath.2015.02.007

[9] Lobov, I. B. et al. WNT7b mediates macrophage-induced programmed cell death in patterning of the vasculature. *Nature* **437**, 417–421 (2005). 16163358 10.1038/nature03928 PMC4259146

[10] Nayak, G. et al. Developmental vascular regression is regulated by a Wnt/beta-catenin, MYC and CDKN1A pathway that controls cell proliferation and cell death. *Development* **145**, baz046 (2018). 10.1242/dev.154898 PMC6031408 29777010

[11] Lang, R., Lustig, M., Francois, F., Sellinger, M. & Plesken, H. Apoptosis during macrophage-dependent ocular tissue remodelling. *Development* **120**, 3395–3403 (1994). 7821211 10.1242/dev.120.12.3395

[12] Watson, E. C. et al. Apoptosis regulates endothelial cell number and capillary vessel diameter but not vessel regression during retinal angiogenesis. *Development* **143**, 2973–2982 (2016). 27471260 10.1242/dev.137513

[13] Franco, C. A. et al. Dynamic endothelial cell rearrangements drive developmental vessel regression. *PLoS Biol.* **13**, e1002125 (2015). 25884288 10.1371/journal.pbio.1002125 PMC4401640

[14] Stenman, J. M. et al. Canonical Wnt signaling regulates organ-specific assembly and differentiation of CNS vasculature. *Science* **322**, 1247–1250 (2008). 19023080 10.1126/science.1164594

[15] Cho, C., Smallwood, P. M. & Nathans, J. Reck and Gpr124 are essential receptor cofactors for Wnt7a/Wnt7b-specific signaling in mammalian CNS angiogenesis and blood-brain barrier regulation. *Neuron* **95**, 1056-1073 e1055 (2017). 28803732 10.1016/j.neuron.2017.07.031 PMC5586543

[16] Zhou, Y. & Nathans, J. Gpr124 controls CNS angiogenesis and blood-brain barrier integrity by promoting ligand-specific canonical wnt signaling. *Dev. Cell* **31**, 248–256 (2014). 25373781 10.1016/j.devcel.2014.08.018 PMC4223636

[17] Vallon, M. et al. A RECK-WNT7 receptor-ligand interaction enables isoform-specific regulation of Wnt bioavailability. *Cell Rep.* **25**, 339-349 e339 (2018). 30304675 10.1016/j.celrep.2018.09.045 PMC6338448

[18] Cullen, M. et al. GPR124, an orphan G protein-coupled receptor, is required for CNS-specific vascularization and establishment of the blood-brain barrier. *Proc. Natl. Acad. Sci. U. S. A.* **108**, 5759–5764 (2011). 21421844 10.1073/pnas.1017192108 PMC3078373

[19] Monvoisin, A. et al. VE-cadherin-CreERT2 transgenic mouse: A model for inducible recombination in the endothelium. *Dev. Dyn.* **235**, 3413–3422 (2006). 17072878 10.1002/dvdy.20982

[20] Percie du Sert, N. et al. The ARRIVE guidelines 2.0: Updated guidelines for reporting animal research. *PLoS Biol.* **18**, e3000410 (2020). 32663219 10.1371/journal.pbio.3000410 PMC7360023

[21] Lizen, B., Claus, M., Jeannotte, L., Rijli, F. M. & Gofflot, F. Perinatal induction of Cre recombination with tamoxifen. *Transgenic Res.* **24**, 1065–1077 (2015). 26395370 10.1007/s11248-015-9905-5

[22] Hao, Y. et al. Dictionary learning for integrative, multimodal and scalable single-cell analysis. *Nat. Biotechnol.* **42**, 293–304 (2024). 37231261 10.1038/s41587-023-01767-y PMC10928517

[23] Wu, T. et al. clusterProfiler 4.0: A universal enrichment tool for interpreting omics data. *Innovation* **2**, 100141 (2021). 34557778 10.1016/j.xinn.2021.100141 PMC8454663

[24] Franzen, O., Gan, L. M. & Bjorkegren, J. L. M. PanglaoDB: A web server for exploration of mouse and human single-cell RNA sequencing data. *Database (Oxford)* **2019**, baz046 (2019). 30951143 10.1093/database/baz046 PMC6450036

[25] Piera-Velazquez, S. & Jimenez, S. A. Endothelial to mesenchymal transition: Role in physiology and in the pathogenesis of human diseases. *Physiol. Rev.* **99**, 1281–1324 (2019). 30864875 10.1152/physrev.00021.2018 PMC6734087

[26] Kuhnert, F. et al. Essential regulation of CNS angiogenesis by the orphan G protein-coupled receptor GPR124. *Science* **330**, 985–989 (2010). 21071672 10.1126/science.1196554 PMC3099479

[27] Lang, R. A. & Bishop, J. M. Macrophages are required for cell death and tissue remodeling in the developing mouse eye. *Cell* **74**, 453–462 (1993). 8348612 10.1016/0092-8674(93)80047-i

[28] Wang, Y. et al. Interplay of the Norrin and Wnt7a/Wnt7b signaling systems in blood-brain barrier and blood-retina barrier development and maintenance. *Proc. Natl. Acad. Sci. U.S.A.* **115**, E11827–E11836 (2018). 30478038 10.1073/pnas.1813217115 PMC6294914

[29] Bergers, G. & Song, S. The role of pericytes in blood-vessel formation and maintenance. *Neuro Oncol.* **7**, 452–464 (2005). 16212810 10.1215/S1152851705000232 PMC1871727

[30] Posokhova, E. et al. GPR124 functions as a WNT7-specific coactivator of canonical beta-catenin signaling. *Cell Rep.* **10**, 123–130 (2015). 25558062 10.1016/j.celrep.2014.12.020 PMC4331012

[31] Xu, Q. et al. Vascular development in the retina and inner ear: Control by Norrin and Frizzled-4, a high-affinity ligand-receptor pair. *Cell* **116**, 883–895 (2004). 15035989 10.1016/s0092-8674(04)00216-8

[32] Baffert, F. et al. Cellular changes in normal blood capillaries undergoing regression after inhibition of VEGF signaling. *Am. J. Physiol. Heart Circ. Physiol.* **290**, H547-559 (2006). 16172161 10.1152/ajpheart.00616.2005

[33] Prakash, V. et al. Ribosome biogenesis during cell cycle arrest fuels EMT in development and disease. *Nat. Commun.* **10**, 2110 (2019). 31068593 10.1038/s41467-019-10100-8 PMC6506521

[34] Morin, C. et al. Intricate ribosome composition and translational reprogramming in epithelial-mesenchymal transition. *Proc. Natl. Acad. Sci. U. S. A.* **121**, e2408114121 (2024). 39636864 10.1073/pnas.2408114121 PMC11648652

[35] Aisagbonhi, O. et al. Experimental myocardial infarction triggers canonical Wnt signaling and endothelial-to-mesenchymal transition. *Dis. Model. Mech.* **4**, 469–483 (2011). 21324930 10.1242/dmm.006510 PMC3124051

[36] Beyer, C. et al. Beta-catenin is a central mediator of pro-fibrotic Wnt signaling in systemic sclerosis. *Ann. Rheum. Dis.* **71**, 761–767 (2012). 22328737 10.1136/annrheumdis-2011-200568 PMC3951949

[37] Wang, S. H. et al. Tumour cell-derived WNT5B modulates in vitro lymphangiogenesis via induction of partial endothelial-mesenchymal transition of lymphatic endothelial cells. *Oncogene* **36**, 1503–1515 (2017). 27593938 10.1038/onc.2016.317

[38] Schafer, C. M. et al. An inhibitor of endothelial ETS transcription factors promotes physiologic and therapeutic vessel regression. *Proc. Natl. Acad. Sci. U.S.A.* **117**, 26494–26502 (2020). 33020273 10.1073/pnas.2015980117 PMC7584886

