# Supplementary material for: GPR124 regulates hyaloid blood vessel regression and is associated with endothelial-mesenchymal transition
Source: Sci Rep. 2026 Apr 29;16:13765. doi: 10.1038/s41598-026-50835-1 (PMC13128929; doi:10.1038/s41598-026-50835-1)
Supplement: Supplementary file 1 — Supplementary Information 1. [file 41598_2026_50835_MOESM1_ESM.pdf]

Figure S1

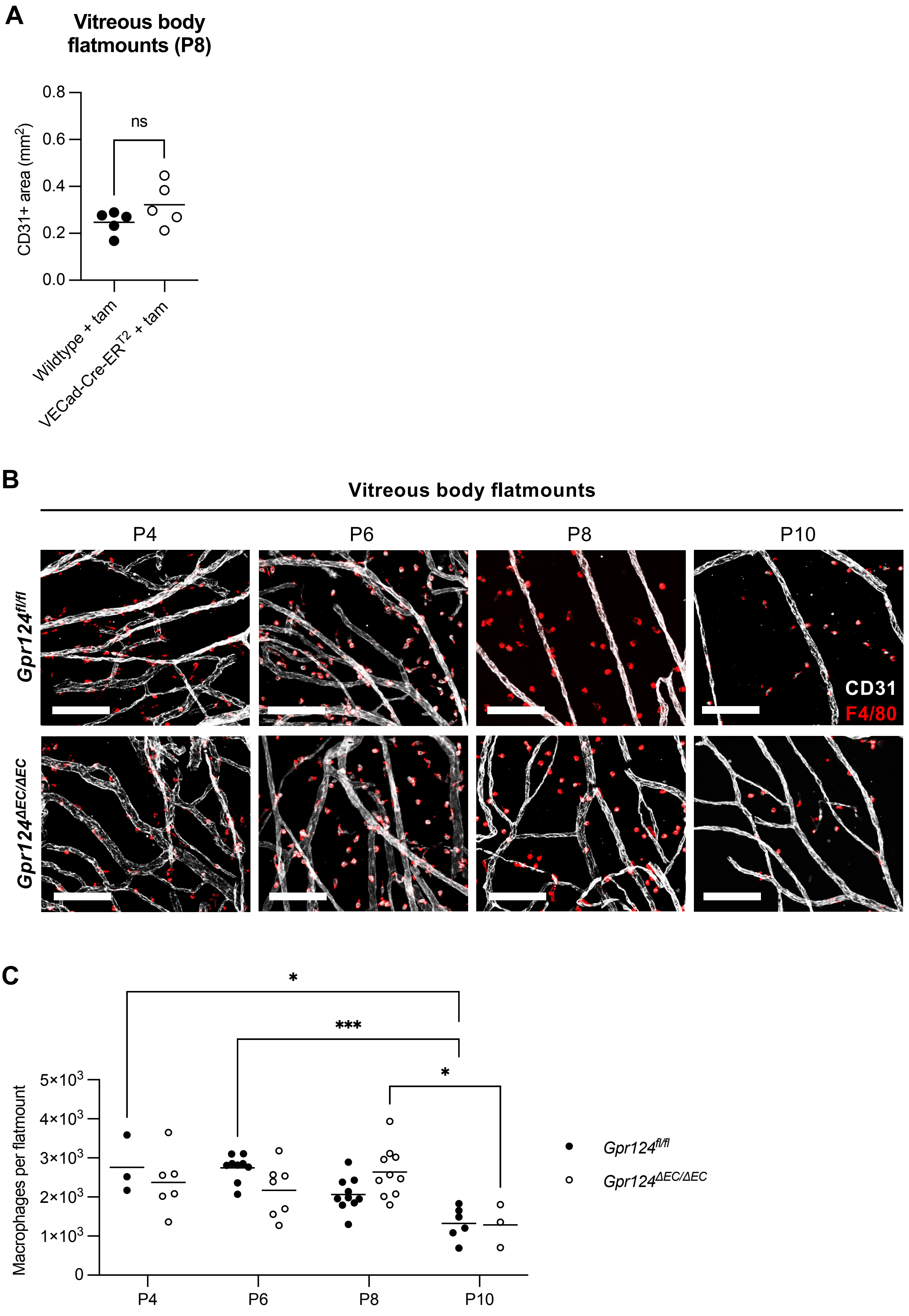

A
Vitreous body flatmounts (P8)
CD31+ area (mm2)
0.8
0.6
0.4
0.2
0.0
ns
Wildtype + tam
VECad-Cre-ERT2 + tam
B
Vitreous body flatmounts
P4
P6
P8
P10
Gpr124fl/fl
Gpr124ΔEC/ΔEC
CD31
F4/80
C
Macrophages per flatmount
5×103
4×103
3×103
2×103
1×103
0
*
***
*
P4
P6
P8
P10
Gpr124fl/fl
Gpr124ΔEC/ΔEC

Figure S1. The VECad-Cre-ER^{T2} transgene does not affect hyaloid vessel regression, and endothelial GPR124 does not regulate hyaloid macrophage numbers. Related to Figure 1.

(A) Quantification of hyaloid vessel regression (CD31-positive area) in vitreous body flatmounts from P8 wildtype vs VECad-Cre-ER^{T2} transgenic mice treated with tamoxifen (P1-P4). Horizontal lines represent mean values (n = 5 mice). ns, not significant (unpaired t test). VECad-Cre-ER^{T2}, vascular endothelial cadherin promoter-driven Cre recombinase fused to a tamoxifen-responsive estrogen receptor; tam, tamoxifen.

(B) CD31 and F4/80 co-immunofluorescence staining of vitreous body flatmounts from *Gpr124*$^{fl/fl}$ and *Gpr124*$^{\Delta EC/\Delta EC}$ mice at indicated postnatal days. Scale bar: 50 µm.

(C) Quantification of F4/80-positive macrophage number in biological replicates of (B). Horizontal lines represent mean values (n ≥ 3 mice). $^{*}p < 0.05$, $^{***}p < 0.001$ (two-way ANOVA with Šídák's post-hoc test).

Figure S2

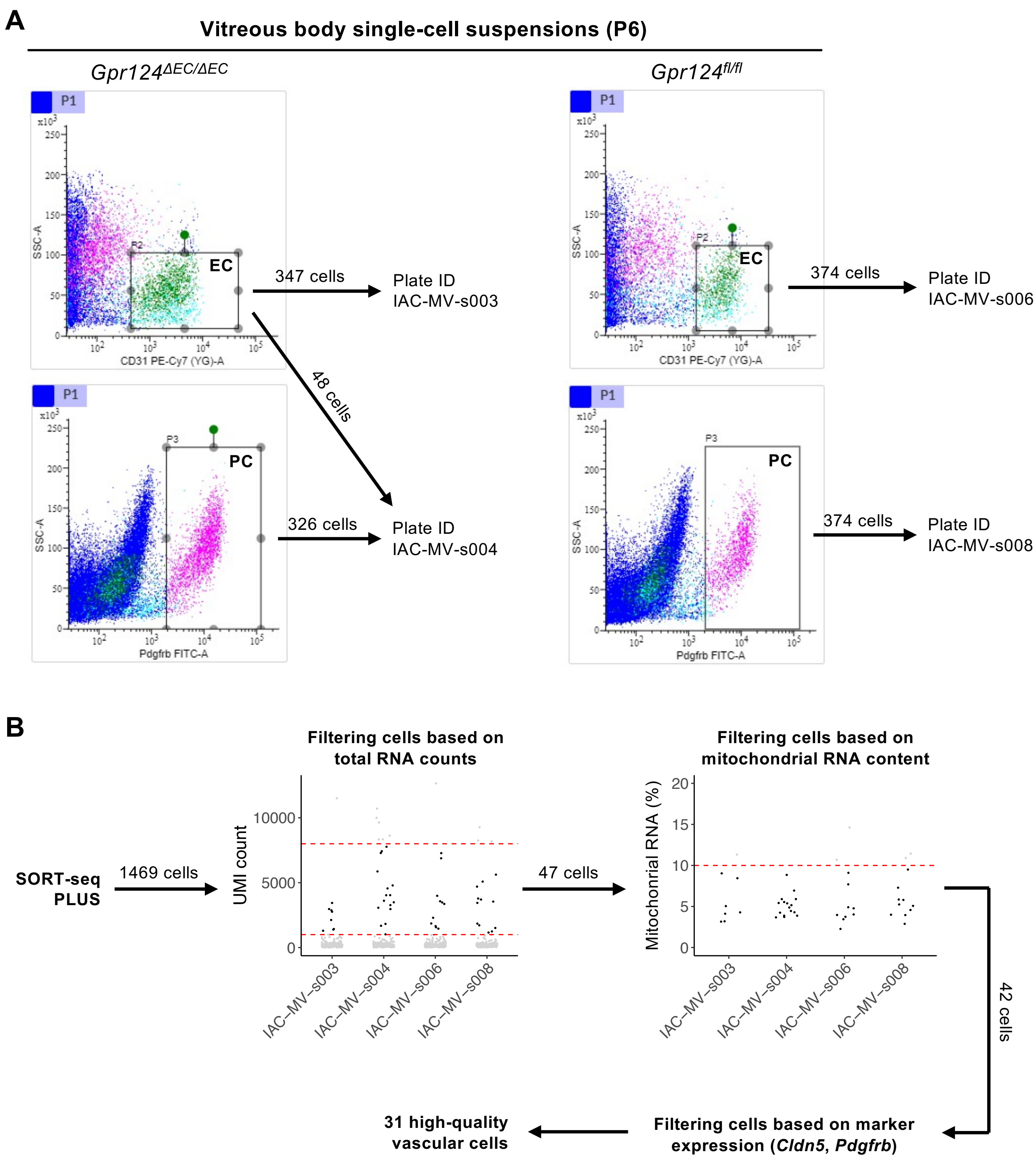

A
Vitreous body single-cell suspensions (P6)
Gpr124ΔEC/ΔEC
Gpr124fl/fl
P1
EC
PC
CD31 PE-Cy7 (YG)-A
Pdgfrb FITC-A
SSC-A
347 cells
Plate ID
IAC-MV-s003
48 cells
326 cells
Plate ID
IAC-MV-s004
374 cells
Plate ID
IAC-MV-s006
374 cells
Plate ID
IAC-MV-s008
B
SORT-seq PLUS
1469 cells
Filtering cells based on total RNA counts
UMI count
47 cells
Filtering cells based on mitochondrial RNA content
Mitochondrial RNA (%)
IAC–MV–s003
IAC–MV–s004
IAC–MV–s006
IAC–MV–s008
42 cells
Filtering cells based on marker expression (Cldn5, Pdgfrb)
31 high-quality vascular cells

Figure S2 (continued)

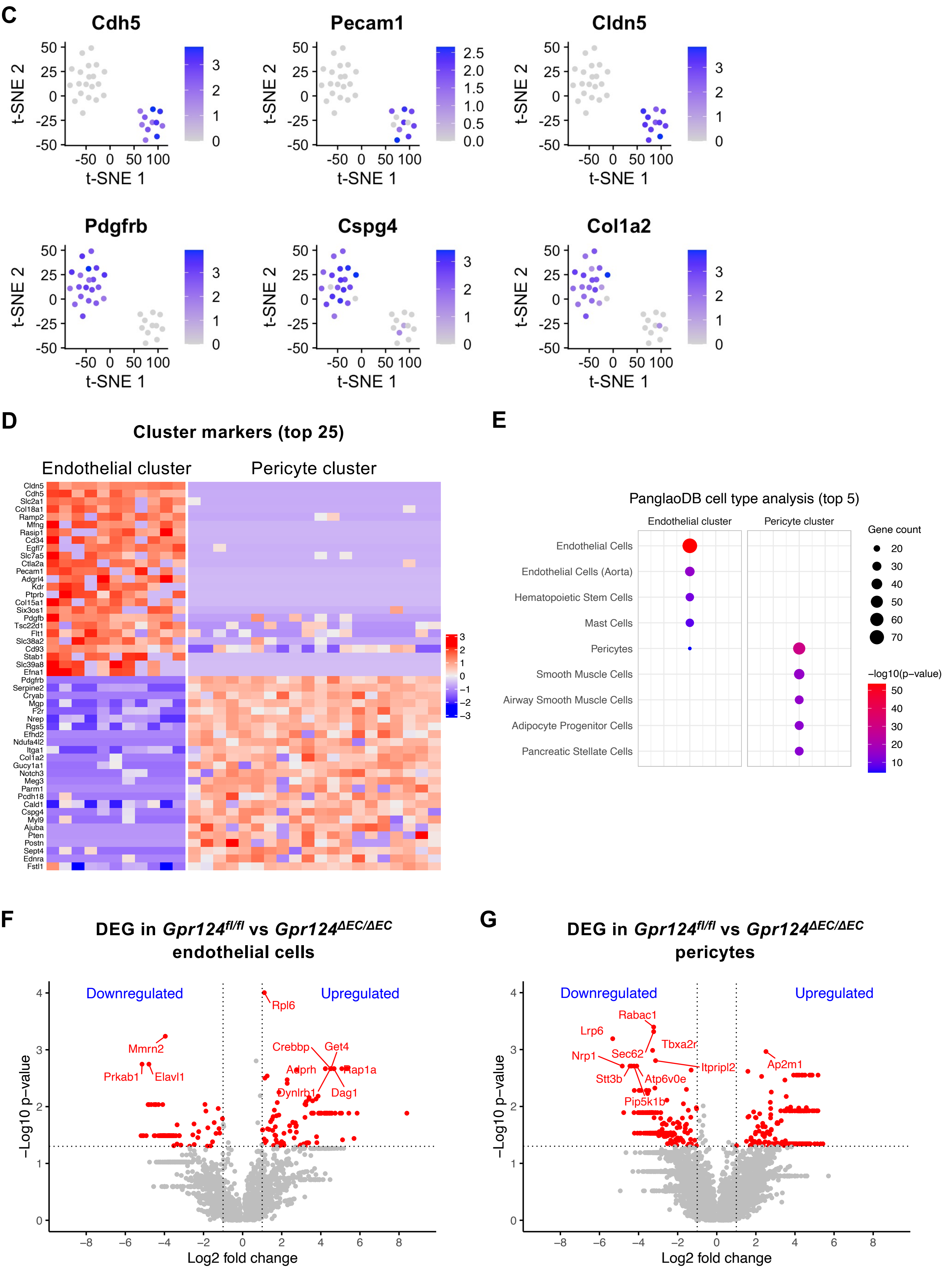

Figure S2 (continued)

H

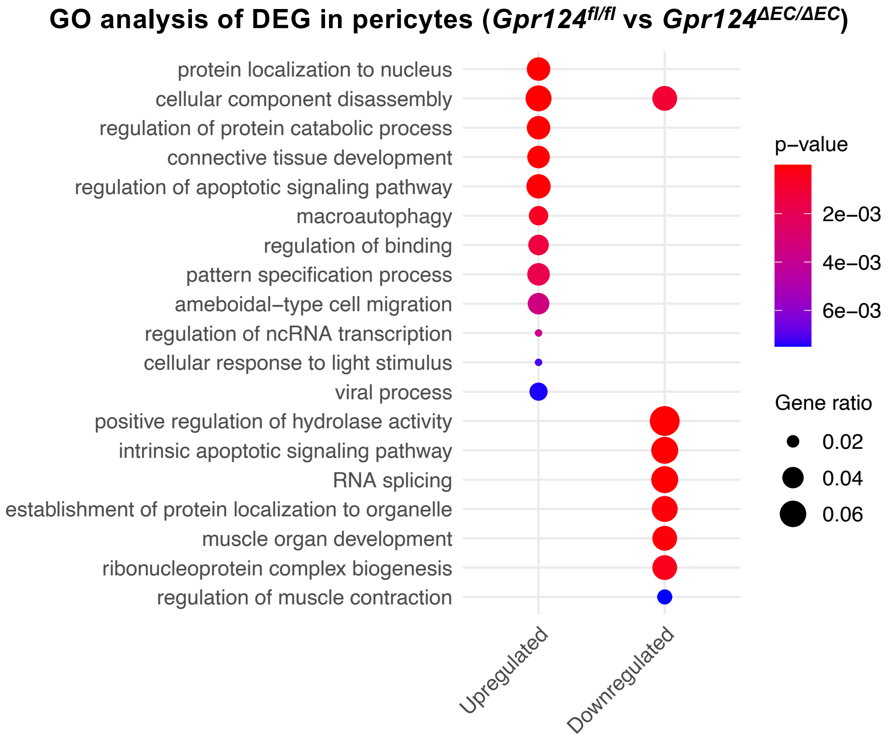

Figure S2. Single-cell RNA sequencing analysis of P6 hyaloid blood vessels. Related to Figure 5.

(A) FACS-based sorting of hyaloid endothelial cells (EC) and pericytes (PC) into 384-well SORT-seq cell capture plates (Single Cell Discoveries). Vitreous body single-cell suspensions from seven *Gpr124*$^{fl/fl}$ and nine *Gpr124*$^{\Delta EC/\Delta EC}$ P6 mice were fluorescently labeled with anti-CD31-PE-Cy7 and anti-PDGFRB-FITC antibodies and subjected to FACS.

(B) Filtering steps for the cells sequenced by the SORT-seq PLUS protocol (Single Cell Discoveries). For marker-based filtering, cells expressing (UMI count > 0) *Cldn5* or *Pdgfrb* were retained. Cells expressing neither marker or both markers were excluded. UMI, unique molecular identifier.

(C) t-SNE plots of sequenced cells showing color-coded mRNA expression (log-normalized UMI counts) of indicated endothelial (top) and pericyte markers (bottom). Blue indicates high expression; gray indicates undetectable expression. t-SNE, t-distributed stochastic neighbor embedding.

(D) Heatmap (scaled UMI counts) illustrating the top 25 differentially expressed genes (by p-value) between the endothelial and pericyte clusters. Complete results are provided in Table S1. Red = upregulated, blue = downregulated or not detected.

(E) PanglaoDB cell type analysis using all markers from the indicated clusters. The top five identified cell types (by p-value) are shown. Complete results are provided in Table S1. Gene count (circle size) indicates the number of matching genes.

(F-G) Volcano plots depicting differentially expressed genes (red dots) between P6 *Gpr124*$^{fl/fl}$ vs *Gpr124*$^{\Delta EC/\Delta EC}$ hyaloid endothelial cells **(F)** or pericytes **(G)**. The top ten differentially expressed genes (by p-value) are labeled. Differential expression criteria: absolute log2 fold change > 1 and $p < 0.05$ (logistic regression with likelihood ratio test). DEG, differentially expressed genes.

(H) GO Biological Process analysis of differentially expressed genes between P6 *Gpr124*$^{fl/fl}$ vs *Gpr124*$^{\Delta EC/\Delta EC}$ hyaloid pericytes. Upregulated and downregulated genes were analyzed separately (columns). Redundant and semantically similar terms were consolidated. Complete results are provided in Table S3. Only terms with a minimum overlap of five genes and a $p < 0.01$ (hypergeometric test) are shown. Gene ratio = overlapping genes : input genes. GO, gene ontology.

Figure S3

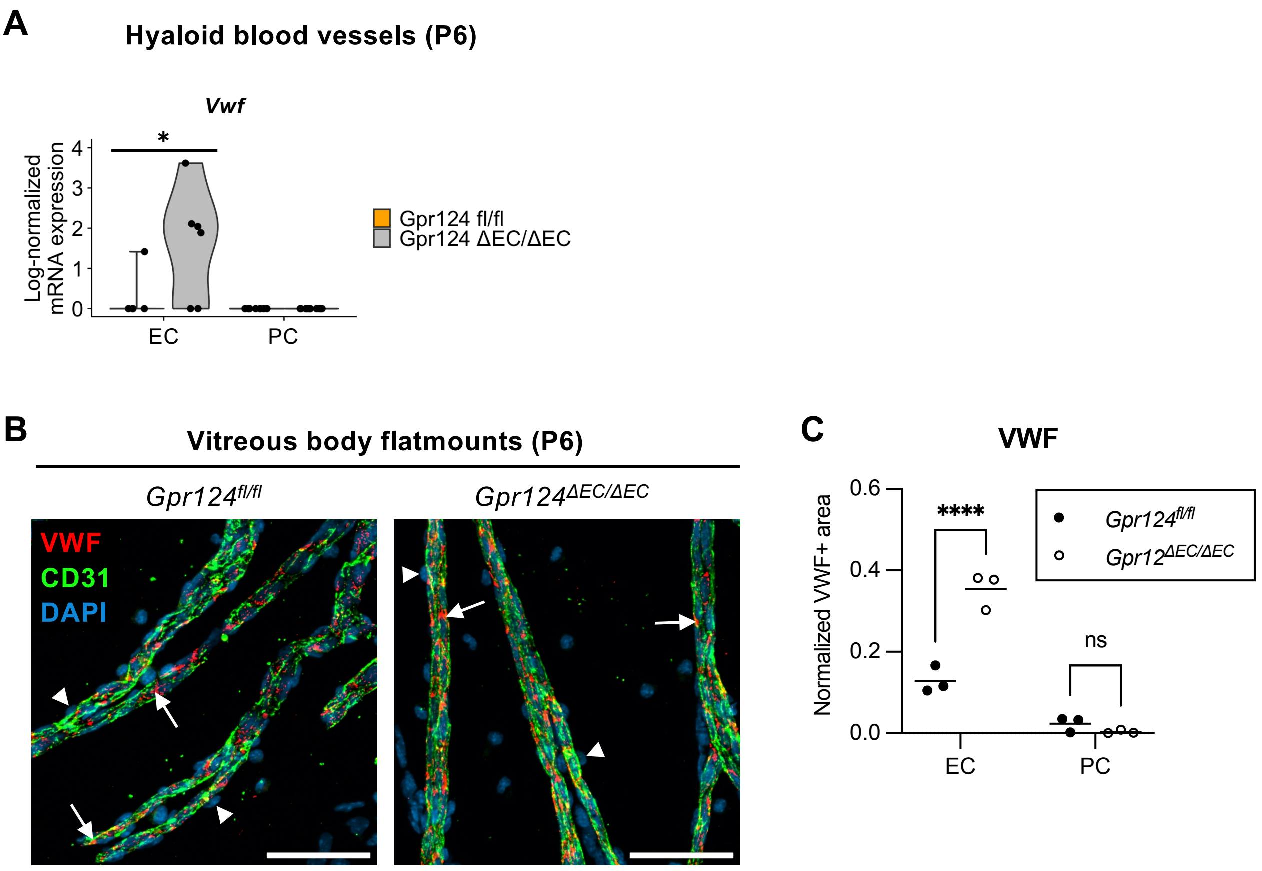

Figure S3. Validation of partial endothelial-mesenchymal transition in hyaloid endothelial cells. Related to Figure 6.

(A) Violin plot depicting log-normalized mRNA expression (UMI counts) of *Vwf* in hyaloid endothelial cells (EC) and pericytes (PC) from P6 *Gpr124*$^{fl/fl}$ vs *Gpr124*$^{ΔEC/ΔEC}$ mice. Dots represent individual cells. *p < 0.05 (logistic regression with likelihood ratio test). Exact p-values are provided in Table S2. UMI, unique molecular identifier; *Vwf*, von Willebrand factor.

(B) VWF and CD31 co-immunofluorescence staining of vitreous body flatmounts from P6 *Gpr124*$^{fl/fl}$ and *Gpr124*$^{ΔEC/ΔEC}$ mice. Arrows denote endothelial cells; arrowheads indicate pericytes. Scale bar: 50 µm.

(C) Quantification of VWF expression in biological replicates of (B). VWF-positive area was normalized to CD31 area in EC and total nuclear area in PC. Horizontal lines show mean values (n = 3 mice). ****p < 0.0001; ns, not significant (two-way ANOVA with Šídák's post-hoc test).

Figure S4

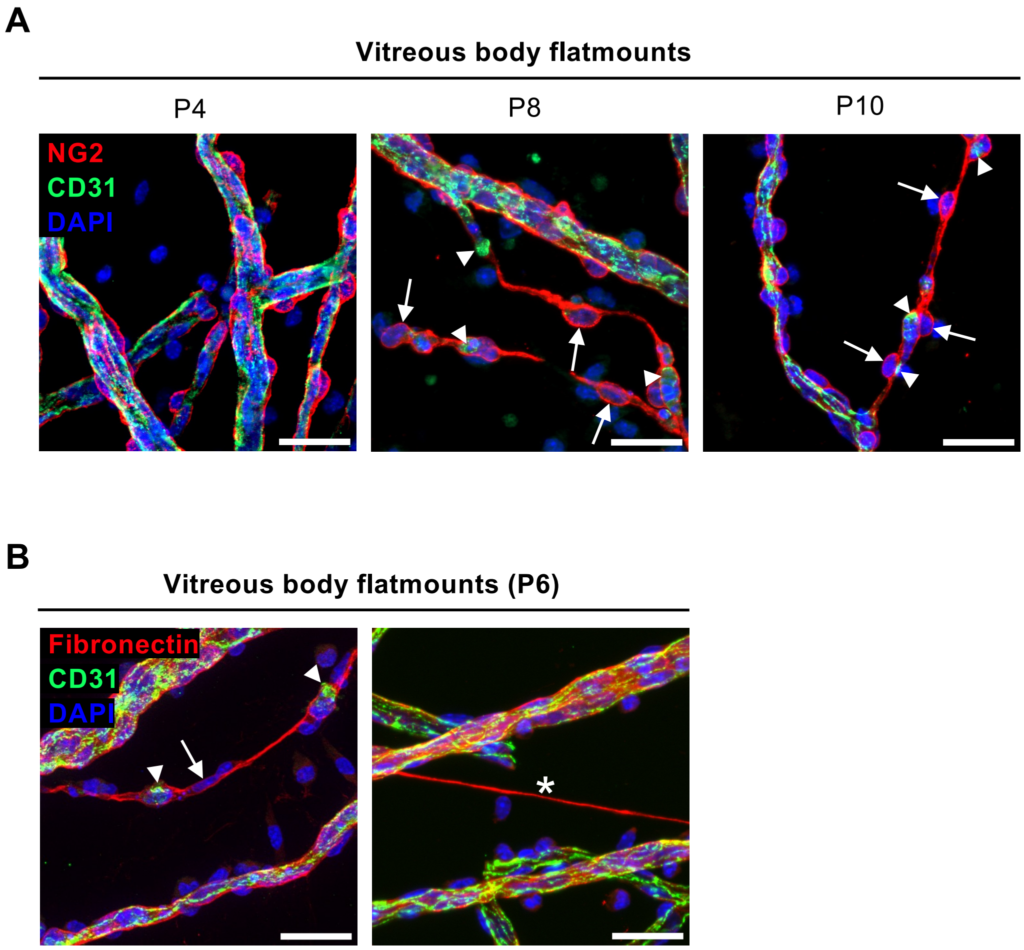

Figure S4. Endothelial cell apoptosis precedes pericyte loss in hyaloid vessel regression
(A) NG2 and CD31 co-immunofluorescence staining of vitreous body flatmounts from mice at indicated postnatal days. Arrows: pericytes within regressing vessels; arrowheads: apoptotic endothelial cells. Scale bar: 25 µm.
(B) Fibronectin and CD31 co-immunofluorescence staining of vitreous body flatmounts from P6 mice. Arrow: pericyte within a regressing vessel; arrowheads: apoptotic endothelial cells; asterisk: acellular regressed vessel. Scale bar: 25 µm.
